# CT-defined body composition is associated with postoperative burst abdomen in patients undergoing laparotomy

**DOI:** 10.1186/s12893-026-03819-x

**Published:** 2026-05-14

**Authors:** Hans-Jonas Meyer, Daniele Romeo, Tihomir Dermendzhiev, Sigmar Stelzner, Uwe Scheuermann, Timm Denecke, Stefan Niebisch, Matthias Mehdorn

**Affiliations:** 1 Department of Diagnostic and Interventional Radiology, University of Leipzig, Liebigstr. 20, Leipzig, 04103 Germany; 2 Department of Biomedicine, Neuroscience and Advanced Diagnostics (BiND), University of Palermo, Palermo, Italy; 3 Department of Visceral, Transplant, Thoracic and Vascular Surgery, University of Leipzig, Leipzig, Germany

**Keywords:** Body composition, Burst abdomen, Computed tomography, Sarcopenia

## Abstract

**Background:**

Postoperative abdominal wall dehiscence (AWD) or burst abdomen (BA) is a relevant complication after abdominal surgery that causes additional surgical procedures, prolonged hospital stays and long-term morbidity. Several underlying risk factors exist and have been described in literature and consist of surgical and medical factors. Recently, CT-derived body composition is of rising interest to provide new prognostic factors in surgical patients. The present study aims to explore the association between CT-defined body composition and postoperative BA.

**Materials and methods:**

A database of patients who underwent abdominal surgery and developed post-operative wound infections in our institution between 2015 and 2018, was assembled. The subgroup of patients with BA was compared to a control group without BA. CT-defined body composition was evaluated in L3-level measuring skeletal muscle index (SMI) for sarcopenia assessment, visceral adipose tissue (VAT) and subcutaneous adipose tissue (SAT). Clinical risk factors and CT-defined body composition were used to predict the occurrence of postoperative BA using discriminatory and binary logistic regression analyses.

**Results:**

A total of 118 patients, 92 (78%) with BA and 26 (22%) without BA were included in the analysis. CT derived body composition parameters for visceral obesity and sarcopenia showed statistically significant differences between the two cohorts. Patients with burst abdomen showed higher VAT (157.6 cm² vs. 84.9 cm², *p* = 0.001) and a significantly lower SMI (46.9 cm²/m² vs. 53.8 cm²/m², *p* = 0.016). Consequently, visceral obesity and sarcopenia were significantly more frequent in patients with BA (*p* = 0.02 and 0.01, respectively). In the multivariable Firth’s penalized logistic regression, visceral obesity (OR = 4.87, 95% CI 1.32–21.91 *p* = 0.02), sarcopenia (OR = 5.94, 95% CI 1.65–26.68 *p* = 0.006), intestinal resection (OR = 9.33, 95% CI 2.33–55.65 *p* < 0.001) and length of the surgical wound (OR = 1.12, 95% CI 1.04–1.22 *p* = 0.001) were independently associated with the occurrence of burst abdomen.

**Conclusion:**

CT-defined body composition with sarcopenia and visceral obesity are strongly associated with postoperative BA. This analysis should be further acknowledged as a potentially important risk factor in surgical care and could aid in clinical decision making.

## Introduction

Burst abdomen (BA) or abdominal wall dehiscence (AWD), is an acute postoperative complication after abdominal surgery that is defined by the dehiscence of all abdominal wall layers with subsequent protrusion of intraabdominal contents through the gap. BA shows a frequency between 1 and 6.6% depending on several surgical factors such as performed surgical procedure and patient’s characteristics [1, 2].

Several different risk factors are known comprising surgical site infection (SSI), poor nutritional status [3], chronic steroid use [3], chronic pulmonary disease and diabetes mellitus [4]. Although complete BA is relatively agile to diagnose, incomplete or subcutaneous BA might be problematic to identify [5]. BA constitutes indication for operative approach and several complications have been reported, such as prolonged hospital stay, bowel evisceration, high incidence of incisional hernia and subsequent reoperation [6].

It is clinically important to identify patients at risk for BA. Presumably, imaging modalities can help to characterize the muscle quantity and quality of the abdominal muscles.

Computed tomography (CT)-defined body composition assessment has shown promising results in several surgical fields. Clear association between CT-defined visceral obesity with postoperative complications was demonstrated in a large meta-analysis comprising a total of 19 studies [7]. This study could show the relationship between visceral obesity with increased surgical site infection, pneumonia, and postoperative pancreatic fistula [7].

In major hepatobiliary surgery, a clear demonstration for the prognostic impact of CT-defined sarcopenia metrics on postoperative complications was already demonstrated [8]. However, moderate heterogeneity existed and cutoffs for the used skeletal muscle thresholds to define sarcopenia varied throughout the included patient samples.

In another recent study, a surrogate diameter defined by CT-images was used to predict BA in a preliminary study [9]. However, it remains unclear how important the whole-cross sectional image assessment of sarcopenia can be to predict BA. Presumably, there should be more included information regarding the muscle quality using the cross-sectional image approach.

Therefore, the purpose of the present study was to investigate the prognostic relevance of CT-defined body composition with the occurrence of BA in a heterogenous surgical patient cohort undergoing median laparotomy.

## Patients and methods

Ethics Review Board of the University of Leipzig approved this study under the reference “419/18-ek” and it was registered retrospectively in the German register for clinical trials (DRKS, DRKS00019058, registration date 19th December 2019). The need for informed consent and the consent to participate was waived by the ethics review board of the University of Leipzig given to the retrospective study. The research was conducted in accordance with local legislation and the Declaration of Helsinki. The patient cohort was recruited between 2015 and 2018 with prospectively collected data in the patient chart and assessed their risk factors for the development of BA.

A part of the present study cohort was previously investigated with a diameter measurement for visceral obesity and for the definition of the rectus muscle [9, 10].

Mass suturing technique with a running suture of PDS loop of the suture strength 1 (Ethicon, Johnson&Johnson, Norderstedt, Germany) was used to perform abdominal wall closure and no prophylactic meshes have been used at that time.

An institutional database of patients who underwent abdominal surgery and developed post-operative wound infections in our institution between 2015 and 2018, was assembled. BA was defined clinically as a partial or complete dehiscence of abdominal wall occurring within the post-operative period and requiring reoperation. SSI was diagnosed according to the Centers for Disease Control definition [11].

The BA group was reconsidered in this study as interventional cohort (IC). To establish a control cohort (CC), we selected 26 patients out of the previously established wound register cohort from an anonymized datasheet who had undergone midline laparotomy, through simple random sampling. These patients had developed SSI, but no BA, and were selected in the same timeframe. The ratio between the two groups was determined through power calculations (clinicalc.com) and based on preliminary measurements regarding visceral obesity (alpha error 0.05; power 0.8). The objective was to include the whole burst abdomen cohort with available CT imaging. Only patients undergoing median laparotomy were included into the CC, as it is known to cause more wound complications opposed to other/smaller incisions.

### Skeletal muscle quantification

CT images were obtained in portalvenous contrast enhanced phase during clinical routine work-up with suspicion of inflammation or tumor staging. All CT were acquired on a 128-slice CT scanner (Ingenuity 128, Philips, Hamburg, Germany). Body composition parameters were semiautomatically determined using the open-source ImageJ software 1.48v (National Institutes of Health Image program). Skeletal muscle areas were calculated on the level of L3 including, psoas muscle, paraspinal muscles and the abdominal wall muscles. The muscle area was semiautomatically measured using the HU threshold levels of -29 and 150 HU, as proposed in similar studies [12, 13]. The skeletal muscle area was divided by the height squared to calculate the skeletal muscle index (SMI). For sarcopenia definition, the SMI threshold proposed by Prado et al. was 52.4 cm²/m² for male and 38.5 cm²/m² for female was, the threshold value by Fearon et al. was 55 cm^2^/m^2^ for male and 39 cm^2^/m^2^ for female and the threshold value by Martin et al. was 53 cm^2^/m^2^ for BMI ≥ 25; 43 cm^2^/m^2^ for BMI < 25 for male patients and 41 cm^2^/m^2^ for female patients. All of the used cut-off values have sex adjusted values [14–16].

### Fat area quantification

VAT and SAT were calculated on the same level of L3 as the skeletal muscle areas. The fat areas were semiautomatically measured using the HU threshold levels of -190 and − 30 HU. The previously proposed threshold value of 100 cm² was used as a cut-off value to determine visceral obesity regardless of the sex. Then according to Baggerman et al. the VAT Index (VATI in cm^2^/m^2^) was used with the threshold values of ≥ 38.7 cm^2^/m^2^ for males and ≥ 24.9 cm^2^/m^2^ for females [17, 18].

**Fig. 1:**
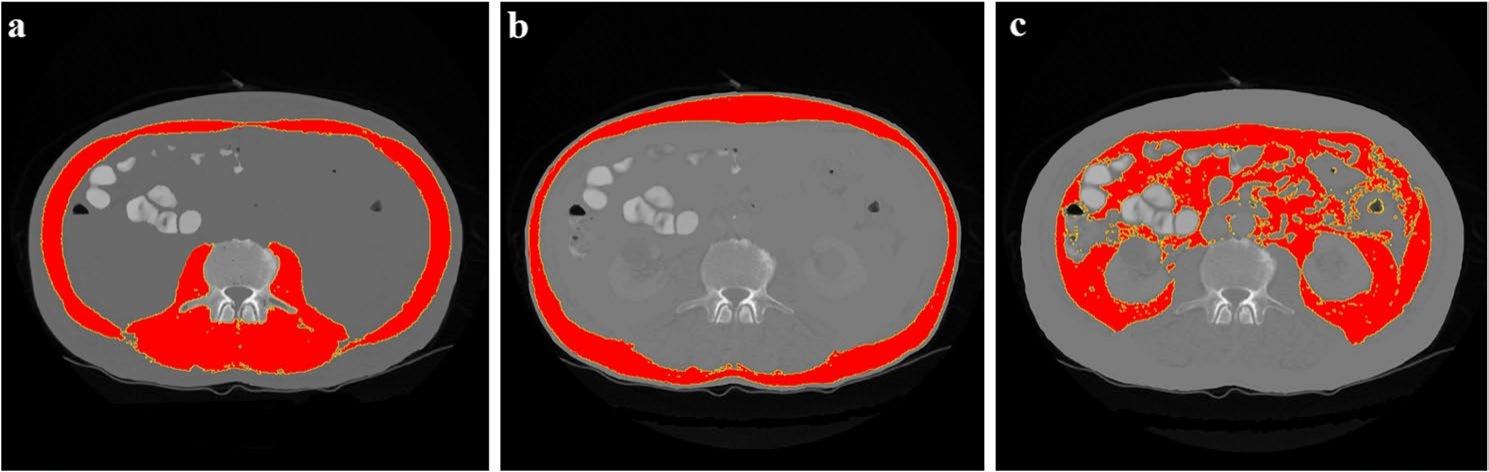
Representative case example of our patient sample. **A** provides the skeletal muscle measurement (**B**) the subcutaneous adipose tissue and **C**) the visceral fat area. The patient had a postoperative burst abdomen

Figure 1 displays a representative case for illustrative purposes of body composition assessment.

### Statistical analysis

The statistical analysis and graphics creation were performed using *R* (version 4.4.3) in ambient RStudio (version 2024.12.1) and SPSS (IBM, Version 25.0; Armonk, NY, USA). Collected data were evaluated by median and interquartile ranges (IQR) of descriptive statistics. For continuous variables, Shapiro-Wilk test was used to assess normal distribution. Discriminations between groups were calculated using Mann-Whitney-U-test and Fisher’s exact test, when suitable. Firth’s penalized logistic regression was used to predict the occurrence of BA in multivariate analysis. Receiver operating characteristic (ROC) curve with the outcome measure of the area under the curve (AUC) was used to assess diagnostic accuracy of the investigated CT-body composition parameters. All parameters that showed a *p*-value < 0.1 in the univariable analyses were included subsequently in a multivariable analysis, together with relevant clinical parameters. In all instances tests were two sided and *P-v*alues < 0.05 were used to demonstrate statistical significance.

## Results

### Patient cohort

A total of 118 patients had abdominal CT imaging within 1 month before the initial surgery and were available for data analysis. Among them, 92 patients developed BA in the post-operative setting, while 26 patients with midline laparotomies and subsequent SSI showed no BA. The patient sample was comprised of 32 (27.1%) female patients and 86 (72.9%) male patients with a median age of 64.4 (IQR 56.2–75.0) years and a median BMI of 26 kg/m^2^ (IQR 22.7–29.3). The demographic overview of the investigated patient sample is provided by Table 1.

**Table 1:** Patient characteristics of the complete cohort, burst abdomen group and the no burst abdomen group. All values are given as absolute number (%) or median value [IQR]. Comparisons between groups were performed with Mann Whitney U test or Fischer exact test, when appropriate. Statistically significant p-values are highlighted in bold.

Variable	Overall cohort	No BA	BA	
	*N* (%) or median [IQR]	*N* (%) or median [IQR]	*N* (%) or median [IQR]	*p*-value
Number of patients	118>	26	92	
Sex				0.315
* Male*	86 (72.9%)	17 (64%)	69 (75%)	
* Female*	32 (27.1%)	9 (36%)	23 (25%)	
Age (years)	64.41 [56.21–75.04]	63.38 [56.44–76.81]	64.96 [56.15–73.94]	0.716
BMI (kg/m²)	26 [22.72–29.37]	26.15 [22.82–28.32]	26 [22.73–29.55]	0.711
Surgical wound characteristics				
* Length (mm)*	16 [11–24]	10 [5–15]	17 [12–25]	**< 0.001**
* Width (mm)*	4 [2–7]	2.25 [1.5–5.5]	4.5 [3–7]	**0.019**
* Depth (mm)*	2 [1–4]	2.5 [1–4]	2 [1.5–4]	0.932
Intestinal resection	43 (36.4%)	4 (15.4%)	39 (42.4%)	**0.012**
Visceral Obesity Baggerman	82 (69.5%)	13 (50%)	69 (75%)	**0.028**
Visceral Obesity Yang	80 (67.8%)	11 (42.3%)	69 (75%)	**0.004**
Sarcopenia Prado	51 (43.2%)	5 (19.2%)	46 (50%)	**0.006**
Sarcopenia Fearon	57 (48.3%)	7 (26.9%)	50 (54.3%)	**0.015**
Sarcopenia Martin	45 (38.1%)	4 (15.4%)	41 (44.6%)	**0.006**
Pneumonia	12 (10.2%)	3 (11.5%)	9 (9.8%)	0.725
Kidney failure	53 (44.9%)	8 (30.8%)	45 (48.9%)	0.121
Delirium	12 (10.2%)	2 (7.7%)	10 (10.9%)	1
Bleeding	12 (19%)	1 (11.1%)	11 (20.4%)	1
Heart disease	34 (28.8%)	10 (38.5%)	24 (26.1%)	0.229
Cirrhosis	18 (15.3%)	1 (3.8%)	17 (18.5%)	0.118
Renal disease	33 (28%)	5 (19.2%)	28 (30.4%)	0.586
Diabetes mellitus	21 (17.8%)	4 (15.4%)	17 (18.5%)	1
Dementia	3 (2.5%)	1 (3.8%)	2 (2.2%)	0.529
Malignancy	69 (58.5%)	16 (61.5%)	53 (57.6%)	0.823
Chemotherapy	29 (24.6%)	7 (26.9%)	22 (23.9%)	0.798
Rheumatic pathology	19 (16.1%)	2 (7.7%)	17 (18.5%)	0.238
Emergency setting	58 (49.2%)	9 (34.6%)	49 (53.3%)	0.121

*Abbreviations*: *VAT *visceral adipose tissue, *SAT *Subcutaneous adipose tissue, *VATI *visceral adipose tissue index, *SMI *skeletal muscle index, *BMI *Body mass index

### CT-derived parameters comparison

The comparison between the two cohorts revealed that VAT and was statistically different between patients with BA and without (84.9 cm² vs. 157.6 cm², *p* = 0.001). SMI was statistically significantly lower in the BA group (53.8 cm²/m² vs. 46.8 cm²/m², *p* = 0.016). Contrary, the SMA was not statistically different (*p* = 0.09). No statistically significant differences were identified for SAT between the two groups. The results are shown in Table 2.

**Table 2:** Discrimination analysis between the BA and non-BA group using the CT-body composition parameters. Statistically significant p-values are highlighted in bold.

CT-Variable	No BA (median)	No BA (IQR)	BA (median)	BA (IQR)	*p* value
*SMA*	153.55	127.99–218.86	145.01	116.12–175.85	0.0984
*VAT*	84.89	66.2–160.36	157.63	104.47–259.02	**0.0015**
*SAT*	144.14	98.87–219.02	176.28	115.85–252.2	0.1295
*VATI*	28.41	22.77–50.33	53.37	34.36–89.33	**0.0014**
*SMI*	53.82	47.21–66.92	46.89	41.23–56.73	0.0161

*Abbreviation*: *SMA *skeletal muscle area, *VAT *visceral adipose tissue, *SAT *Subcutaneous adipose tissue, *VATI *visceral adipose tissue index, *SMI *skeletal muscle index, *BA *Burst Abdomen

Using the different sarcopenia threshold values, the frequency of sarcopenic patients was significantly higher in the BA group for every threshold value (*p* = 0.006 using the Prado threshold value, *p* = 0.01 using the Fearon threshold and *p* = 0.006 using the Martin threshold value). Similar results were identified according to the visceral obesity definition by Baggerman et al. and Yang et al. (*p* = 0.02 and *p* = 0.003, respectively) [17, 18].

### Univariate and multivariate logistic regression of risk factors for BA

**Fig. 2:**
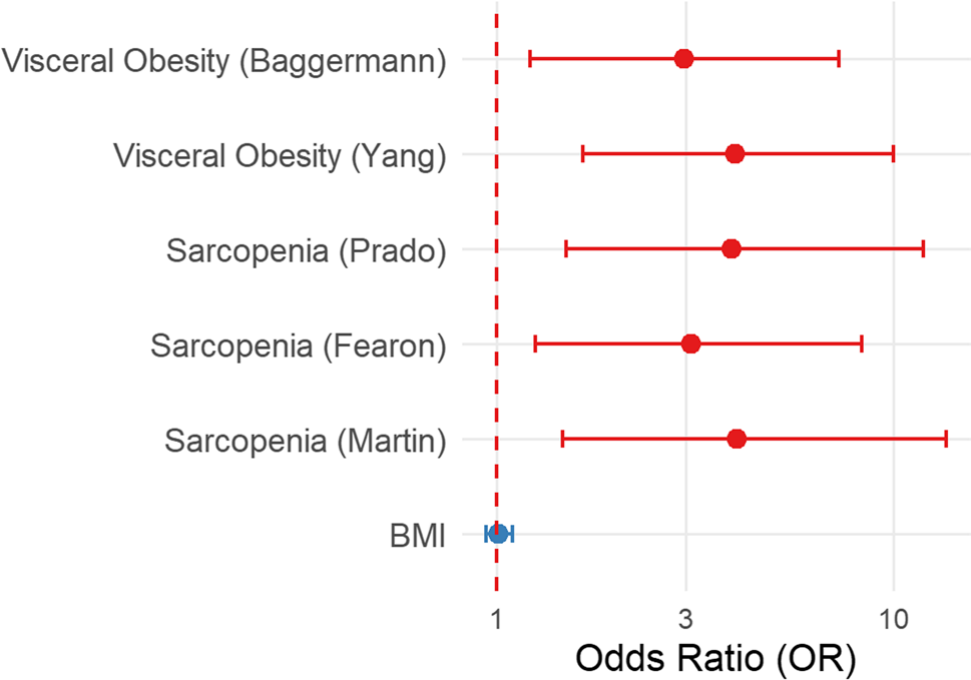
Forest plot of the logistic regression analysis results

The univariate Firth’s penalized logistic regression showed that the CT-derived parameters were associated with the occurrence of burst abdomen. Among the surgical predictors, intestinal resection and the length of the surgical site infection were significantly associated with the dehiscence event (*p* = 0.02 and < 0.0001, respectively). To reduce collinearities among overlapping radiological indices, visceral obesity was defined by the Baggerman threshold value. For sarcopenia, the threshold proposed by Prado et al. was included as it was widely used, in order to enhance comparability to the precedent literature. In the multivariate Firth´s penalized logistic regression model, parameters with *p*-value < 0.1 were included, resulting in the inclusion of visceral obesity, sarcopenia, intestinal resection, wound length, kidney failure or renal disease and emergency setting and cirrhosis. In parallel, acknowledged risk factors such as BMI, age, diabetes mellitus were retained in the model. The following tested parameters showed significance in the multivariate analysis: visceral obesity (OR = 4.87, 95% CI 1.32–21.91 *p* = 0.02), sarcopenia (OR = 5.94 95% CI 1.65–26.68 *p* = 0.006), intestinal resection (OR = 9.33 95% CI 2.33–55.65 *p* < 0.001) and length of the surgical site infection (OR = 1.12 95% CI 1.04–1.22 *p* = 0.001). The corresponding results are shown in Table 3. Figure 2 shows the corresponding Forest plot.

**Table 3:** Univariate and multivariate logistic regression analysis to predict burst abdomen. Statistically significant p-values are highlighted in bold.

UNIVARIATE analysis					MULTIVARIATE analysis				
Variable	OR	CI lower	CI upper	*p* value	Variable	OR	CI lower	CI upper	*p* value
SMA	0.991	0.982	1	**0.048**					
VAT	1.009	1.003	1.016	**<0.001**					
SAT	1.004	0.999	1.009	0.107					
VATI	1.026	1.009	1.047	**0.001**					
SMI	0.959	0.928	0.989	**0.008**					
Visceral Obesity Baggermann	2.957	1.216	7.263	**0.017**	Visceral Obesity Baggermann	4.871	1.317	21.907	**0.017**
Visceral Obesity Yang	3.986	1.644	9.966	**0.002**					
Sarcopenia Prado	3.909	1.492	11.898	**0.005**	Sarcopenia Prado	5.942	1.649	26.68	**0.006**
Sarcopenia Fearon	3.089	1.253	8.319	**0.014**					
Sarcopenia Martin	4.029	1.462	13.585	**0.006**					
BMI	1.01	0.938	1.098	0.893	BMI	1.002	0.894	1.134	0.976
Age	0.992	0.96	1.023	0.616	Age	0.98	0.938	1.022	0.349
Age > 60	1.079	0.437	2.581	0.866					
Sex	0.587	0.235	1.52	0.266					
Intestinal resection	3.692	1.338	12.454	**0.01**	Intestinal resection	9.33	2.332	55.648	**< 0.001**
Wound Length (mm)	1.154	1.076	1.257	**< 0.001**	Wound Length (mm)	1.12	1.043	1.223	**0.001**
Wound Width (mm)	1.101	0.982	1.285	0.107					
Wound Depth (mm)	0.989	0.78	1.285	0.927					
Emergency	2.096	0.877	5.27	0.097	Emergency	0.827	0.225	2.957	0.769
Pneumonia	0.764	0.221	3.256	0.691					
Kidney Failure or renal disease	2.509	1.054	3.256	**0.038**	Kidney Failure or renal disease	1.292	0.383	4.367	0.676
Delirium or dementia	1.043	0.317	4.332	0.949					
Bleeding	1.498	0.228	15.091	0.658					
Heart disease	0.562	0.23	1.411	0.215					
Cirrhosis	3.94	0.915	36.902	0.068	Cirrhosis	7.581	0.752	167.147	0.089
Diabetes Mellitus	1.159	0.396	4.043	0.798	Diabetes Mellitus	0.527	0.108	2.484	0.409
Malignancy	0.862	0.35	2.05	0.739					
Chemotherapy	0.83	0.324	2.288	0.701					
Rheumatological disease	2.272	0.648	11.983	0.216					

All the statistically significant parameters in the univariate analysis were included in the multivariate analysis

*Abbreviations*: *VAT *visceral adipose tissue, *SAT *Subcutaneous adipose tissue, *DM *Diabetes Mellitus

### Sensitivity analysis

To assess robustness of our findings, sensitivity analyses were conducted. For the multivariate analysis, Firth’s penalized logistic regression was conducted with cut-offs proposed by Fearon et al. and Martin et al. The resulting odds ratios were consistent with the multivariate Firth’s penalized logistic regression in the reported model. For the cut-off proposed by Fearon et al., an OR = 4.59 (95%CI: 1.16–22.28, *p* = 0.02), while sarcopenia assessed through the cut-off proposed by Martin et al., showed OR = 5.32 (95% CI: 1.49–23.43, *p* = 0.009).

### Diagnostic accuracy

To assess diagnostic accuracy of the CT-body composition a ROC analysis was performed.

**Fig. 3:**
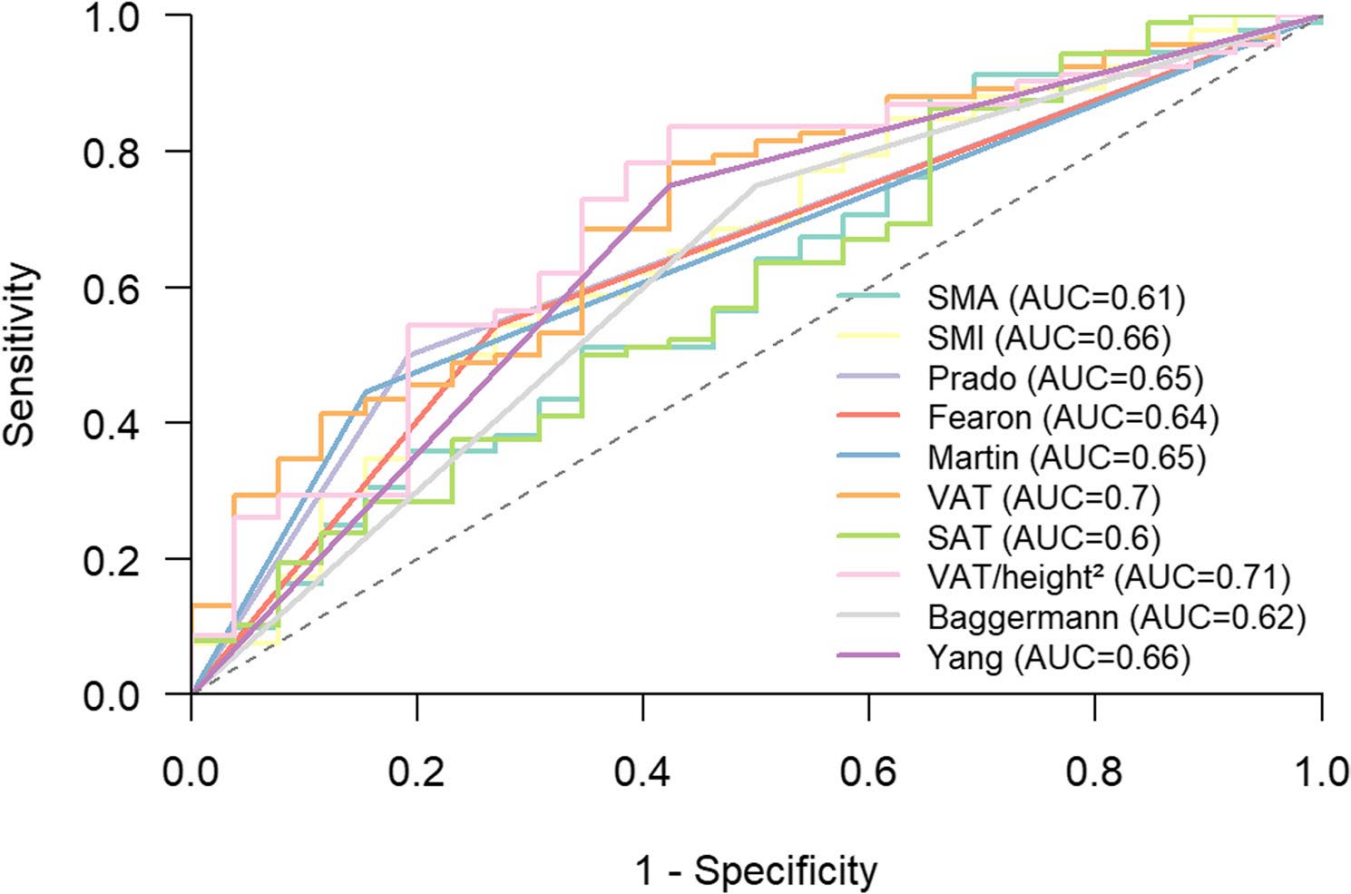
ROC curve of the investigated CT-body composition parameters to predict postoperative burst abdomen. The highest AUC achieved the visceral adipose tissue index with 0.71

The highest AUC value achieved the VATI with 0.71 (95% CI: 0.59–0.82). For the skeletal muscle parameters, the sarcopenia definition by Prado et al. achieved an AUC of 0.65 (95% CI: 0.56–0.74). Overall, the investigated CT-body composition parameters achieved a moderate accuracy. Figure 3 provides the corresponding ROC curves of the parameters.

## Discussion

The present study can demonstrate a clear association between CT-derived sarcopenia and visceral obesity as one major predictive risk factor for the development of post-operative abdominal fascial dehiscence. One key finding is that sarcopenia, among imaging-derived body composition parameters, shows the strongest association with the occurrence of BA.

In literature, clinical and patient specific factors and characteristics have been investigated to predict post-operative abdominal wall dehiscence [19, 20]. However, the translation into clinical routine is limited due to large number of parameters used in these scores and difficult measurement techniques.

Combined clinical and imaging-based risk scores could enhance clinical care as they could potentially identify patients with postoperative complications and change the clinical decision process.

Therefore, the possibility of BA prediction by the inclusion of CT-defined body composition parameters could be taken in consideration in further studies, to increase the diagnostic accuracy but also reduce some clinical factors in the scoring systems. For the occurrence of BA, it seems logical that mechanical properties of different intraabdominal tissues seem relevant as a risk factor.

CT-defined body composition, compared to simple BMI measurements, is able to provide more comprehensive information regarding body compartments. It is therefore needed to evaluate new imaging-based factors as prognostic factors for BA.

In our results we could demonstrate that even in multivariable analysis sarcopenia and visceral obesity remained significant with a stronger association for the sarcopenia with the occurrence of BA. This clearly shows that both known risk factors of the CT-defined body composition are significant predictors of BA.

However, as demonstrated by the diagnostic accuracy analysis, the predictive value of the investigated CT-body composition parameters, showed only a moderate value. For a stronger predictive value, the sarcopenia assessment should be incorporated in a multivariable risk score. This should be investigated in future trials.

Pathophysiologically, visceral obesity increases the intraabdominal pressure and subsequent the tension on the abdominal fascial layers, while sarcopenia could be a reflection of nutritional status, able to have a direct effect on tissues characteristics.

Different studies with small cohorts show diverging results regarding body composition parameters and the risk for burst abdomen, with reported associations between visceral obesity and occurrence of BA [21] but also negative studies without any identified association [13].

One important aspect of CT-body composition studies is that different threshold values and methods are used throughout the literature studies. The proposed gold standard for the measurement of body composition compartments is one axial CT slice of the level of the third vertebra (L3) [22, 23]. However, there are also studies using one slice at the umbilicus level [24]. Some authors did not investigate area measurements but provided diameter, such as anterior aspect of the vertebral body to the linea alba and the skin [25].

Throughout the published literature it was highlighted that associations between obesity parameters and either SSI [22, 24, 25 ] or other obesity related diseases exist.

The present study is now the first representative systematic analysis to provide insight into the associations between CT-defined body composition analysis using a methodologically timely approach and the occurrence of BA.

Another key finding of the present analysis is that sarcopenia was a stronger predictor than visceral obesity, highlighting the importance of the skeletal muscle structure in the development of BA. This is in good agreement with the literature highlighting the skeletal muscle assessment via imaging in various different fields in medicine.

A recent study has demonstrated that CT measurements of the abdominal wall including the linea alba was also associated with the occurrence of BA with a high diagnostic accuracy [10].

The current results are in line with the published literature that low-skeletal muscle mass as indication of sarcopenia is an important risk factor for patients undergoing major surgical procedures. One could deduce that imaging markers derived from CT images could be incorporated into surgical treatment planning and for better risk assessment. However, we want to emphasize that, before any clinical translation, these findings need to be investigated in larger, multicentre study as well as in cohorts without SSI.

The present analysis has several limitations. The retrospective single center study design is prone to potential inherent bias. In particular, an imbalance in subgroup sizes may affect the statistical power of the analysis and could lead to an overestimation of effect sizes, larger confidence intervals are registered and it may indicate model instability. There also may be some selection bias in the current patient sample. In particular, all patients had SSI, which constitutes a clinically relevant high-risk subgroup. Therefore, the study design itself may introduce collider bias and it may limit the generalizability of the findings, potentially reflecting associations specific to the high-risk group rather than the general laparotomy population. These aspects underscore the need for multicenter, larger studies, in order to confirm the present findings. Furthermore, body composition quantification was performed by a single reader in a semiquantitative manner, and no inter-reader agreement was evaluated. Although this may introduce, it is likely to be minimal, as it has already been shown that CT body composition can be reliably measured with high inter-reader agreement.

## Conclusion

CT-defined body composition may aid to identify patients at risk for a postoperative burst abdomen. Sarcopenia showed a stronger association than visceral obesity. The inclusion of these CT-defined parameters into clinical care as preoperative imaging markers is recommended although further investigations are needed.

## Data Availability

The datasets generated are available from the corresponding author uponreasonable request.
